# FBXO6-mediated RNASET2 ubiquitination and degradation governs the development of ovarian cancer

**DOI:** 10.1038/s41419-021-03580-4

**Published:** 2021-03-25

**Authors:** Mei Ji, Zhao Zhao, Yue Li, Penglin Xu, Jia Shi, Zhe Li, Kaige Wang, Xiaotian Huang, Bin Liu

**Affiliations:** 1 Department of Gynecology, The First Affiliated Hospital of Zhengzhou University, Zhengzhou, China; 2 Jiangsu Key Laboratory of Marine Pharmaceutical Compound Screening, College of Pharmacy, Jiangsu Ocean University, Lianyungang, China

**Keywords:** Oncogenes, Ubiquitin ligases

## Abstract

RNASET2 (Ribonuclease T2) functions as a tumor suppressor in preventing ovarian tumorigenesis. However, the mechanisms underlying the regulation of RNASET2 protein are completely unknown. Here we identified the F-box protein FBXO6, a substrate recognition subunit of an SCF (Skp1-Cul1-F-box protein) complex, as the ubiquitin E3 ligase for RNASET2. We found that the interaction between FBXO6 and RNASET2 induced RNASET2 instability through the ubiquitin-mediated proteasome degradation pathway. FBXO6 promoted K48-dependent ubiquitination of RNASET2 via its FBA domain. Through analysis of the TCGA dataset, we found that FBXO6 was significantly increased in ovarian cancer tissues and the high expression of FBXO6 was related to the poor overall survival (OS) of ovarian cancer patients at advanced stages. An inverse correlation between the protein levels of FBXO6 and RNASET2 was observed in clinic ovarian cancer samples. Depletion of FBXO6 promoted ovarian cancer cells proliferation, migration, and invasion, which could be partially reversed by RNASET2 silencing. Thus, our data revealed a novel FBXO6-RNASET2 axis, which might contribute to the development of ovarian cancer. We propose that inhibition of FBXO6 might represent an effective therapeutic strategy for ovarian cancer treatment.

## Introduction

Ovarian cancer is one of the most common malignant tumors of the female reproductive system and has become a serious medical and social problem^1^. Ninety percent of ovarian malignant tumors are epithelial ovarian cancer. Surgery combined with platinum-based chemotherapy is currently the main treatment method for ovarian cancer, but the treatment effect is still unsatisfactory^2^. The 5-year survival rate has always hovered between 25% and 30%, and the chemotherapy resistance is the main reason for treatment failure. Unfortunately, the molecular etiology of ovarian cancer remains elusive^3^.

Ribonucleases are a class of nucleases that can hydrolyze RNA to 3’-single nucleotides by using 2’, 3’-cyclic nucleotides^4^. Ribonucleases can be divided into RNase T1 family and RNase T2 family based on their molecular mass^4^. RNase T2 is widely present in microorganisms, animals, and plants in nature^5^. RNASET2 is a RNase T2 enzyme that exists in the human body and it is also the only extracellular nuclease of this family^6^. RNASET2 has been identified as a tumor suppressor gene with reduced expression in primary ovarian tumors^7^, melanoma^8^, and non-Hodgkin’s lymphoma^9^. Interestingly, its tumor suppressive effect was not related to its enzymatic activity^7^. Induced overexpression of RNASET2 could inhibit ovarian tumorigenesis^10^. On the contrary, knockdown of RNASET2 was able to stimulate ovarian tumor growth in nude mice^7^. However, it is still unclear how RNASET2 protein is regulated in ovarian cancer.

CRL (Cullin-RING Ligase) is the most abundant type of multi-subunit ubiquitin ligase in the human body. It consists of Cullin, Ring protein, and substrate recognition subunits, and can regulate the degradation of many tumor-related proteins in cells. Seven members of the Cullin family have been reported, including Cullin1, Cullin2, Cullin3, Cullin4A, Cullin4B, Cullin5, and Cullin7, and each Cullin member could form a CRL complex with other components^11^. The F-box protein is the critical component of the CRL1 or SCF (SKP1/Cullin1/F-box protein) protein complex. Nearly 70 F-box proteins have been identified in human. F-box proteins can recruit substrates through protein–protein interaction, to promote substrates ubiquitination and degradation^12^. According to the C-terminal secondary structure, F-box proteins can be divided into three classes, including the FBXW, the FBXL, and the FBXO. One of the most prominent features of the F-box protein family’s substrate recognition is that they can specifically recognize phosphorylated substrates^13^. However, in FBXO, there is another subfamily containing FBA domains, including FBXO2, FBXO6, FBXO17, FBXO27, and FBXO44. This subfamily can specifically recognize glycoproteins through the FBA domain^14^. FBXO6 is widely distributed and expressed in most human tissues and cells^14^. FBXO6 has been reported to target DNA damage checkpoint kinase Chk1 for destruction to the arrest cells at the S phase and plays a role in chemotherapy resistance^15^. Consistent with the role of FBXO6 in cell cycle control, we also found that FBXO6 is a cell cycle-specific phosphorylated protein, which is phosphorylated during mitosis and dephosphorylated after the cell enters the G1 phase^16^. FBXO6 can bind to Mad2 and BubR1 proteins to inactivate the spindle checkpoint, indicating that FBXO6 might have a function similar to tumor-promoting genes^16^. It is worth noting that FBXO6 can recognize glycosylated proteins through its FBA domain and participate in the regulation of endoplasmic reticulum-related glycoprotein degradation^17^. Indeed, we have previously found that FBXO6 can target glycosylated Ero1L protein for destruction to regulate endoplasmic reticulum stress-induced cell apoptosis^18^. Moreover, through large-scale immunoprecipitation/mass spectrometry (IP/MS) assay, we have previously discovered and verified many glycoproteins that were associated with FBXO6 protein^19^. However, the majority of FBXO6 substrates are still unidentified.

Here we found that FBXO6 was overexpressed in ovarian cancer tissues and the high expression of FBXO6 was associated with the poor survival of ovarian cancer patients at advanced stages. At the molecular level, FBXO6 recognized and targeted RNASET2 protein for proteasomal-dependent destruction through its FBA domain. Our data further revealed a critical role of the FBXO6-RNASET2 axis in the regulation of ovarian cancer cell proliferation, migration, invasion in vitro, and tumor formation in nude mice in vivo.

## Material and methods

### Cell culture and tissue samples

HEK293T, human ovarian cancer cells A2780, and OVCAR-3 were obtained from American Type Culture Collection with and tested for mycoplasma contamination. HEK293 and A2780 cells were cultured in high-glucose Dulbecco’s modified Eagle’s medium (DMEM; Invitrogen, CA, USA) containing 10% fetal bovine serum (FBS) at 37 °C in 5% CO_2_. OVCAR-3 cells were grown in RPMI medium (Invitrogen, CA, USA) containing 10% FBS at 37 °C in 5% CO_2_. Eighty-eight paraffin-embedded specimens of human ovarian cancer were obtained from the First Affiliated Hospital of Zhengzhou University and with appropriate patient consent. Immunohistochemical (IHC) staining of the paraffin-embedded tumor tissues was performed using anti-FBXO6 (sc-134339, Santa Cruz, USA) and anti-RNASET2 (sc-393729, Santa Cruz, USA) primary antibodies, and an ABC Elite immunoperoxidase kit according to the manufacturer’s instructions.

### Plasmids

Plasmids containing F-box protein genes were described previously^19^. *RNASET2* gene was amplified from HEK293T cells by PCR reaction and was cloned into pcDNA3.1-HA vector. All plasmids were completely sequenced and transfected into cells by using Lipofectamine 2000 (Invitrogen) according to the manufacturer’s instructions.

### RNA interference, RNA isolation, and real-time PCR

The Lentiviral short hairpin RNAs (shRNAs) against human FBXO6 and RNASET2 were purchased from Merck-Sigma and the target sequences were as follows:

FBXO6-shRNA1: 5′-CCGGCCTACGAAATGTGCCTCAAGTCTCGAGACTTGAGGCACATTTCGTAGGTTTTT-3′;

FBXL6-shRNA2:

5′-CCGGTGTGCTGAAGAGGATATGTTTCTCGAGAAACATATCCTCTTCAGCACATTTTT-3′;

RNASET2-shRNA-1 5′-CCGGCCTGAGACAGTATGCGAGAAACTCGAGTTTCTCGCATACTGTCTCAGGTTTTTG-3′;

RNASET2-shRNA-2 5′-CCGGAGATCGTGGCCCTTCAATTTACTCGAGTAAATTGAAGGGCCACGATCTTTTTTG-3′;

Total RNA of cell lysate was extracted by using TRIzol reagent (Invitrogen, USA). Oligo dT was used to prime cDNA synthesis. Real-time PCR was then performed by using a SYBR Green Premix Ex Taq (TaKaRa, Japan) on Light Cycler480 (Roche, Switzerland). GAPDH was used as internal control. Differences in gene expression were calculated using 2^−ΔΔCt^ method. Primers used for quantitative PCR analysis were as follows: FBXO6 forward, 5′- ATCCTACGAAATGTGCCTCAAG-3′ and reverse, 5′-CCAACACGAAGTAGTCAGCCG-3′; RNASET2 forward, 5′-TGGCCCGATAAAAGTGAAGGA-3′ and reverse, 5′-AACGAGTGAATTACGTCAGGC-3′; GAPDH forward, 5′-ACAACTTTGGTATCGTGGAAGG-3′ and reverse, 5′-GCCATCACGCCACAGTTTC-3′.

### CRISPR/Cas9 knockout cell lines

A2780 cells were transfected with FBXO6 CRISPR/Cas9 KO (h) KO plasmid (sc-405919, Santa Cruz Biotechnology) using Lipofectamine 2000 following the manufacturer’s instructions. A2780 cells were selected with 2 µg/ml puromycin for up to 2 weeks. Single clones were then selected and the knockout (KO) efficiency was verified by western blot assay.

### Western blotting and antibodies

Cells were lysed with lysis buffer (100 mM Tris-HCl pH 6.8, 100 mM dithiothreitol, 1% SDS, 10% glycerol) and boiled for 10 min. Proteins were then separated by 10% SDS-polyacrylamide gel electrophoresis (PAGE) and transferred to polyvinylidene difluoride membrane. Membranes were blocked in 5% non-fat milk in phosphate-buffered saline (PBS) for 1 h before incubation with primary antibodies overnight at 4 °C. Membranes were washed with PBS for three times and incubated with secondary antibody for 1 h at room temperature. Primary antibodies were used as indicated: anti-Flag M2 (1 : 5000 dilution, F1804, Sigma), anti-FBXO6 (1 : 1000 dilution, sc-134339, Santa Cruz, USA), anti-RNASET2 (1 : 1000 dilution, sc-393729, Santa Cruz, USA), anti-SKP1 (1 : 1000 dilution, sc-5281, Santa Cruz, USA), anti-HA (1 : 5000 dilution, H9658, Sigma), anti-K48-linkage Specific Polyubiquitin (1 : 1000 dilution, D9D5, Cell Signaling Technology), anti-K63-linkage Specific Polyubiquitin (1 : 1000 dilution, D7A11, Cell Signaling Technology), and anti-GAPDH (1 : 5000 dilution, sc-47724, Santa Cruz, USA).

### Immunoprecipitation

The IP process has been described previously^20^. Briefly, cells were lysed with IP buffer (100 mM NaCl, 20 mM Tris-HCl pH 8.0, 0.5 mM EDTA, 0.5% (v/v) Nonidet P-40) with protease inhibitor cocktail and phosphorylate inhibitor for 30 min on ice. Cells were sonicated and the lysates were centrifuged. For exogenous IP, the filtered supernatant was first incubated with mouse IgG resin at 4 °C for 30 min to remove the nonspecific binding proteins and then incubated with Flag M2 resin at 4 °C overnight. The resin was washed eight times with IP buffer and two times with PBS plus 200 ng 3× Flag peptides. The final elutes were subjected to western blot analysis. For endogenous IP, the filtered supernatant was incubated with either anti-FBXO6 or anti-RNASET2, or IgG and protein A/G beads overnight at 4 °C in a rotating wheel. Immunoprecipitates were washed three times with IP buffer and two times with PBS. SDS loading buffer was then added and proteins were eluted by boiling at 95 °C for 5 min.

### In vivo ubiquitination assay

The in vivo ubiquitination assay has been described previously^21^. Briefly, 293T cells were transfected with HA-RNASET2 with or without Flag-FBXO6 and His-ubiquitin for 36 h. Cell lysates were sonicated in IP buffer with protease inhibitor cocktail and phosphorylate inhibitor for 10 min on ice. The supernatant was incubated with anti-HA resin overnight at 4 °C in a rotating wheel. The resin was then washed with IP buffer and boiled in SDS loading buffer. Boiled samples were separated by 10% SDS-PAGE and subjected to western blotting with anti-polyubiquitin linkage-specific antibodies.

### Colony formation analysis

One thousand ovarian cancer cells were seeded in a six-well plate per well and then cultured for up to 2 weeks. The numbers of colonies containing more than 50 cells were counted after 0.05% crystal violet staining.

### Cell migration and invasion assays

For migration assay, ovarian cancer cells were inoculated in six-well plates with serum-free medium for 24 h. When the cell confluence reached ~90%, a 20 µl pipette tip was used to scratch the monolayer and then cells were rinsed with PBS to remove non-adhered cells. Images were taken at 0, 24 and 48 h in six random fields for each group. The distance of cell migration was calculated by subtracting the scratch width at 0 h from the scratch width at 24 and 48 h. For invasion assay, 20 μl of Matrigel (Corning) were even plated onto the upper chamber of a Transwell insert (Corning, NY, USA). Ovarian cancer cells (5 × 10^4^) were resuspended with 100 μl of culture medium and seeded into the top chamber. The lower chamber was incubated with culture medium with 10% FBS. Twenty-four hours later, the insert was fixed with 4% paraformaldehyde for 30 min. Non-migrated cells on the top surface of the insert were removed and migrated cells on the lower surface of the insert were stained with 0.05% crystal violet. Images of cells on the Transwell membrane were taken with a microscope at 200× magnification and cell numbers were counted.

### Xenograft assays

Animal study was approved by Animal Care and Use Committee of the First Affiliated Hospital of Zhengzhou University. Four-week-old, male BALB/cA nude mice were purchased from National Rodent Laboratory Animal Resources (Shanghai, China). All mice were kept in a specific pathogen-free facility and randomized into groups. Cells (1 × 10^7^) were suspended in 50 µl of DMEM medium, mixed 1 : 1 with Matrigel and injected into the flanks of male nude mice. Tumor sizes were measured by a caliper and calculated using the formula length × width 2 × 1/2. Tumor weights were measured after mice were killed.

### Statistical analyses

Data are presented as mean ± SD. Statistical analysis was performed with GraphPad Prism 6.0 software. The differences between groups were calculated using the Student’s *t*-test or one-way analysis of variance using a Tukey’s post hoc test. *P* < 0.05 were considered statistically significant.

## Results

### Cullin1-based ubiquitin E3 ligase mediates the degradation of RNASET2 in ovarian cancer cells

**Fig. 1:**
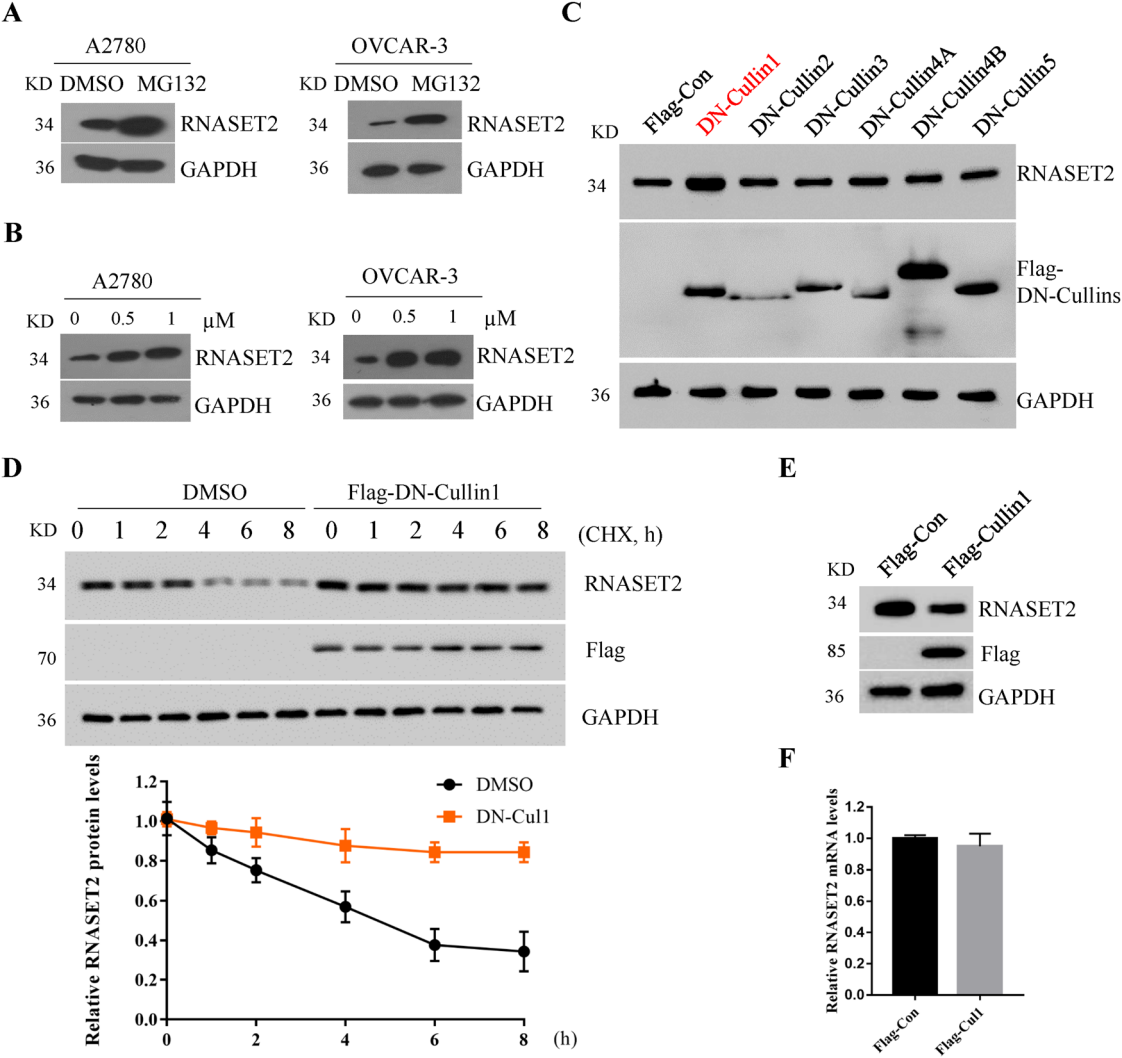
Cullin1-based ubiquitin E3 ligase mediates the degradation of RNASET2 in ovarian cancer cells. **A** Western blot analysis of A2780 and OVCAR-3 cells treated with or without 10 μM MG132 for 4 h. **B** Western blot analysis of A2780 and OVCAR-3 cells treated with an indicated concentration of MLN4924 for 4 h. **C** Western blot analysis of A2780 cells transfected with Flag-tagged DN-Cullin1, DN-Cullin2, DN-Cullin3, DN-Cullin4A, DN-Cullin4B, DN-Cullin5, or Flag-Con vector, respectively. **D** A2780 cells were transfected with Flag-Con or Flag-DN-Cullin1 for 24 h. Cells were then treated with 50 μg/mL CHX for the indicated time. The whole cell lysates (WCLs) were subjected to western blot analysis with indicated antibodies. Statistic results of western blotting analysis were obtained by ImageJ software and normalized to actin GAPDH. Error bars indicate the means ± SD, *n* = 3. **E** Western blot analysis of A2780 cells transfected with Flag-Con or Flag-Cullin1. **F** The mRNA levels of RNASET2 in **E** were determined by real-time PCR assay.

The posttranscriptional regulation of RNASET2 has not been reported yet. Treatment of A2780 or OVCAR-3 cells with the proteasome inhibitor MG132 resulted in the accumulation of RNASET2 protein, suggesting that RNASET2 protein was regulated by the ubiquitin–proteasome system (Fig. 1A). MLN4924 is a small molecule inhibitor of NEDD8-activating enzyme, which can significantly inhibit the neddylation modification of all Cullins to inactivate the entire CRLs, leading to the accumulation of CRL substrates^22^. A2780 or OVCAR-3 cells treated with MLN4924 for 4 h also caused the accumulation of endogenous RNASET2 protein (Fig. 1B), suggesting RNASET2 is a substrate of CRLs in ovarian cancer cells. To further test which Cullin is responsible for the degradation of RNASET2, six dominant negative (DN) Cullin members, including DN-Cullin1, DN-Cullin2, DN-Cullin3, DN-Cullin4A, DN-Cullin4B, and DN-Cullin5 were expressed into 293T cells. As shown in Fig. 1D, among those DN-Cullin members, only DN-Cullin1 could significantly induce the accumulation of endogenous RNASET2 protein (Fig. 1C). Consistent with it, in the presence of DN-Cullin1, the half-life of RNASET2 was significantly extended (Fig. 1D). On the contrary, overexpression of Cullin1 shortened the half-life of RNASET2 (Fig. 1E) without effecting its mRNA level (Fig. 1F). Taken together, these results suggest that the degradation of RNASET2 is governed by SCF E3 ligases.

### FBXO6 interacts with RNASET2 in a FBA domain-dependent manner

**Fig. 2:**
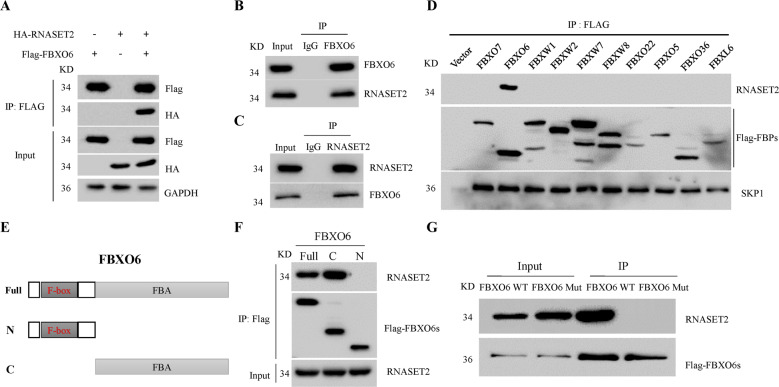
FBXO6 interacts with RNASET2 in a FBA domain-dependent manner. **A** 293T cells were co-transfected with Flag-FBXO6 and HA-RNASET2 for 36 h. Cells were collected and lysed. The WCLs were immunoprecipitated by anti-Flag M2 resin and subjected to western blot analysis as indicated. **B** A2780 cells were treated with MG132 for 4 h. Cells were then collected and lysed. Lysates were immunoprecipitated with anti-FBXO6 antibody and analyzed by immunoblotting as indicated. A nonspecific IgG was used as a negative control. **C** OVCAR-3 cells were treated with MG132 for 4 h. Cells were then collected and lysed. Lysates were immunoprecipitated with anti-RNASET2 antibody and analyzed by immunoblotting as indicated. A nonspecific IgG was used as a negative control. **D** 293T cells were transfected with Flag-Con or the indicated FLAG-tagged F-box protein plasmids (FBPs) for 36 h. Cells were collected and lysed. The WCLs were immunoprecipitated by anti-Flag M2 resin, eluted by 3× Flag peptides and immunoblotting as indicated. **E** The figure represents the domains of the FBXO6 deletion constructs. **F** 293T cells were transfected with the indicated Flag-tagged FBXO6 deletion constructs for 36 h. Cells were collected and lysed. The WCLs were immunoprecipitated by anti-Flag M2 resin, eluted by 3× Flag peptides and immunoblotting as indicated. **G** OVCAR-3 cells were transfected with the Flag-FBXO6 WT or Flag-FBXO6 Mut for 36 h. Cells were collected and lysed. The WCLs were immunoprecipitated by anti-Flag M2 resin, eluted by 3× Flag peptides and immunoblotting as indicated.

Previously, we have identified the potential interacting glycoproteins of FBXO6 generated by IP/MS assay^19^. We found that RNASET2 is one of the putative interacting glycoproteins of FBXO6. Co-IP experiments revealed that Flag-FBXO6 can bind exogenously expressed HA-RNASET2 (Fig. 2A). Importantly, the interaction between endogenous FBXO6 and RNASET2 proteins was also confirmed in both A2780 and OVCAR-3 cells (Fig. 2B, C). To investigate the specificity of this binding, we randomly screened ten F-box proteins. All of these F-box proteins were associated with SKP1, whereas FBXO6 was the only protein that bound to RNASET2 (Fig. 2D). FBXO6 contains an N-terminal F-box domain followed by a C-terminal FBA domain (Fig. 2E). To map the region within FBXO6 protein that mediates its interaction with RNASET2, truncated mutants of FBXO6 were generated. We found that the C-terminal FBA domain of FBXO6 is able to bind to RNASET2 (Fig. 2F). The FBA domain of FBOX6 is required for its glycoprotein-recognized activity and RNASET2 has been documented as a glycoprotein^23^. To test whether FBXO6 recognized glycosylated RNASET2, Flag-FBXO6 wild-type (WT), and Flag-FBXO6 Mut, an FBA domain mutant of FBXO6 without glycoprotein-recognized activity^19^, were transfected into OVCAR-3 cells, respectively. The lysates were subjected to IP with anti-FLAG M2 resin. We found that Flag-FBXO6 WT can effectively recognize endogenous RNASET2, but Flag-FBXO6 Mut has completely lost this ability (Fig. 2G), indicating that FBXO6 recognizes the glycosylated RNASET2 protein.

### FBXO6 negatively regulates the stability of RNASET2

**Fig. 3:**
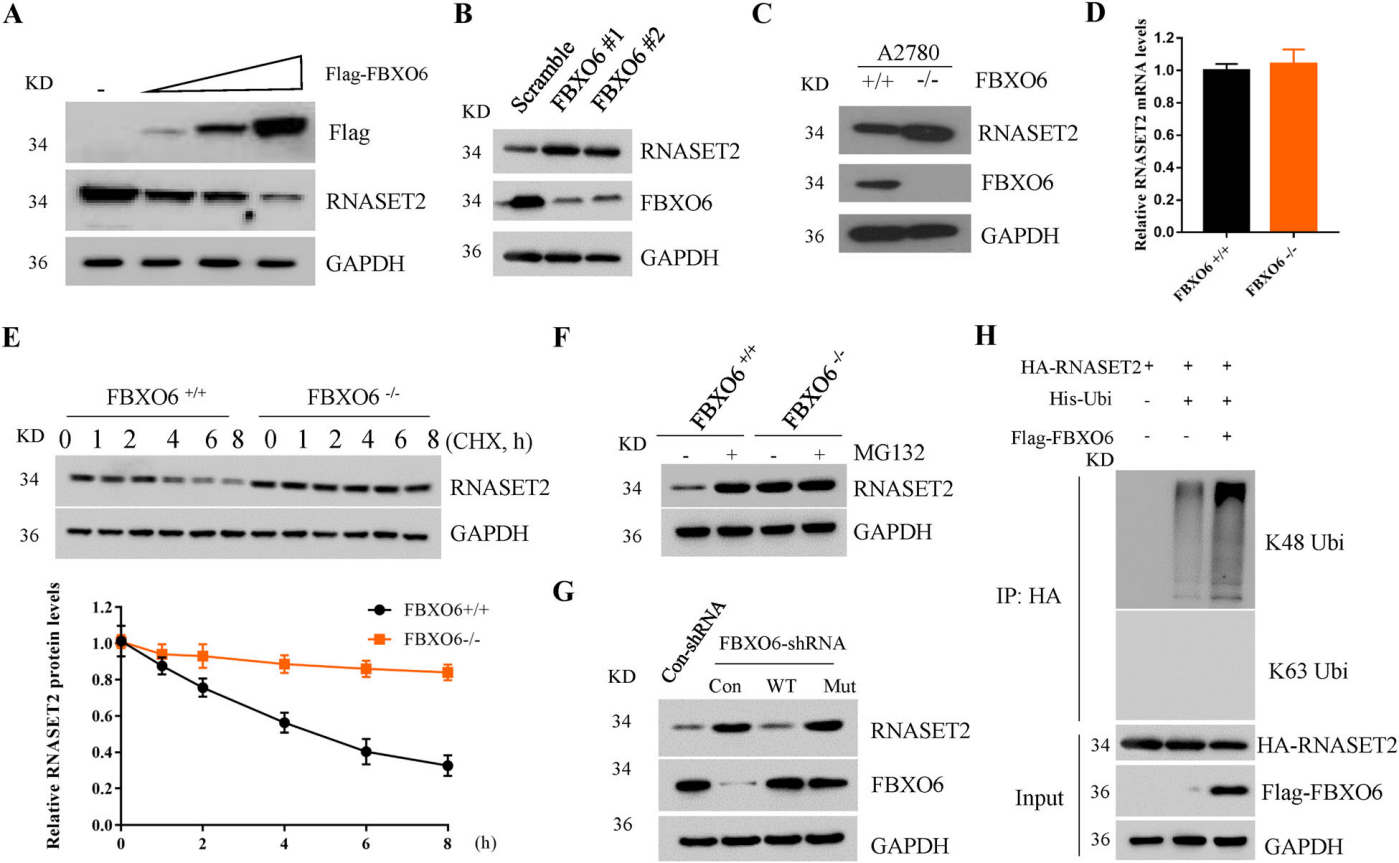
FBXO6 negatively regulates the stability of RNASET2. **A** Western blot analysis of OVCAR-3 cells transfected with Flag-Con or increased Flag-FBXO6 plasmids. **B** Western blot analysis of OVCAR-3 cells transfected with scramble or FBXO6 shRNAs. **C** A2780 FBXO6 knockout (FBXO6^−/−^) cells were generated by CRISPR assay and the WCLs were subjected to western blot analysis with indicated antibodies. **D** The mRNA levels of RNASET2 in **C** were determined by real-time PCR assay. **E** FBXO6^+/+^ and FBXO6^−/−^ A2780 cells were treated with 50 μg/mL CHX for the indicated time. Cells were lysed and the WCLs were subjected to western blot analysis with the indicated antibodies. Statistic results of immunoblotting analysis were obtained by ImageJ software and were normalized to GAPDH intensities. Error bars indicate the means ± SD, *n* = 3. **F** FBXO6^+/+^ and FBXO6^−/−^ A2780 cells were treated with or without 10 μM MG132 for 4 h. Cells were lysed and the WCLs were subjected to western blot analysis with the indicated antibodies. **G** A2780 cells were transfected with con-shRNA or FBXO6-shRNA for 72 h. Cells were then either untreated or transfected with Flag-Con, FBXO6 WT, and FBXO6 Mut for an additional 48 h, respectively. Cells were lysed and the WCLs were subjected to western blot analysis with the indicated antibodies. **H** 293T cells were transfected with HA-RNASET2 with or without Flag-FBXO6 and His-ubiquitin for 36 h. Cells were lysed and the WCLs were immunoprecipitated by anti-HA resin and immunoblotting as indicated.

The interaction between FBXO6 and RNASET2 suggests that SCF-FBXO6 could be the SCF E3 ligase for RNASET2 degradation. To this end, FBXO6 was overexpressed into OVCAR-3 cells to monitor the endogenous RNASET2 protein levels of by immunoblotting. We found that FBXO6 decreased RNASET2 protein expression in a dose-dependent manner, suggesting FBXO6 could accelerate the degradation of RNASET2 protein (Fig. 3A). On the contrary, silencing the expression of FBXO6 by two different shRNAs in OVCAR-3 cells resulted in the accumulation of RNASET2 protein (Fig. 3B). To avoid the off-target effects of shRNAs, we also generated FBXO6^−/−^ A2780 cells by clustered regularly interspaced short palindromic repeats (CRISPR)-based gene editing. We found that RNASET2 protein, but not its mRNA level, was raised in FBXO6^−/−^cells when compared with FBXO6^+/+^ cells. (Fig. 3C, D). Moreover, the half-life of RNASET2 was significantly increased in FBXO6^−/−^ cells (Fig. 3E). FBXO6^−/−^ cells showed high and sustained levels of RNASET2 compared to FBXO6^+/+^ A2780 cells (Fig. 3F). Notably, the FBXO6-mediated proteasomal degradation of RNASET2 confirmed the observation that RNASET2 protein levels were restored only in FBXO6^+/+^ A2780 cells (Fig. 3F). Importantly, restoring the expression of FBXO6 WT, but not FBXO6 Mut, into FBXO6-silenced A2780 cells reduced the high RNASET2 protein level (Fig. 3G), demonstrating FBXO6 recognizes glycosylated RNASET2 for degradation. We then test whether FBXO6 regulates the ubiquitination of RNASET2. 293T cells were transfected with HA-RNASET2 with or without Flag-FBXO6 and His-ubiquitin for 36 h. RNASET2 proteins were immunoprecipitated by anti-HA resin and immunoblotting with anti-polyubiquitin linkage-specific antibodies (Fig. 3H). We found that FBXO6 significantly promoted the K48-linked polyubiquitin modification of RNASET2, but not the K63-linked one, suggesting FBXO6 triggered the degradation signaling of RNASET2 protein. Together, these results indicate that FBXO6 is the SCF E3 ligase for RNASET2 protein destruction.

### FBXO6 regulates ovarian cancer cells proliferation, migration, and invasion

**Fig. 4:**
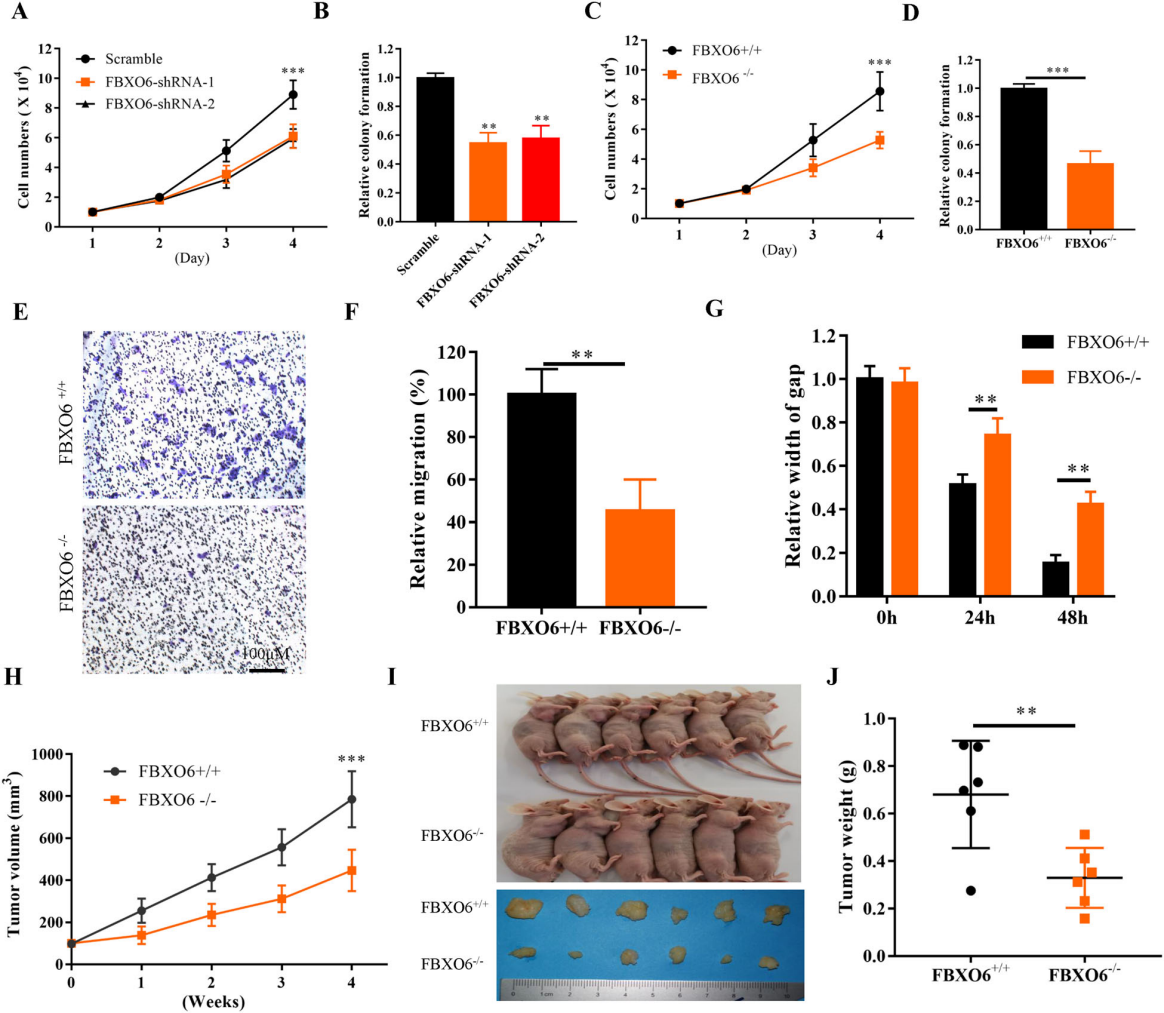
FBXO6 regulates ovarian cancer cells proliferation, migration, and invasion. **A** The cell growth curve of OVCAR-3 cells transfected with scramble or FBXO6 shRNAs. **B** Clonogenic assay of OVCAR-3 cells transfected with scramble or FBXO6 shRNAs. **C** The cell growth curve of FBXO6^+/+^ and FBXO6^−/−^ A2780 cells. **D** Clonogenic assay of FBXO6^+/+^ and FBXO6^−/−^ A2780 cells. **E** Representative images of migrated FBXO6^+/+^ and FBXO6^−^^/−^ A2780 cells in a transwell assay with Matrigel. **F** Quantification of migrated cells in **E**. Data were shown as mean ± SD of three independent experiments. **p < 0.01. **G** The migration of FBXO6^+/+^ and FBXO6^−/−^ A2780 cells in scratch experiments. Data were shown as mean ± SD of three independent experiments. **p < 0.01. **H** FBXO6^+/+^ (1 × 10^7^) and FBXO6^−/−^ A2780 cells were subcutaneously injected into each nude mouse for about 4 weeks. Tumor volumes were detected at the indicated dates. *n* = 6 for each group. ***p < 0.001. **I** The image shows the representative tumor-bearing mice and xenografts for each group. **J** At the end of 4 weeks, mice were killed and the tumor weights were measured. **p < 0.01.

Given the critical roles of RNASET2 in proliferation, migration, and invasion of ovarian cancer^7^, we then test whether FBXO6 was also able to govern these events. We found that silencing the expression of FBXO6 in OVCAR-3 cells by shRNAs can lead to decreased cell growth and colony formation (Fig. 4A, B). These phenomena were also observed in A2780 FBXO6^−/−^ cells compared with FBXO6^+/+^ cells (Fig. 4C, D), suggesting a regulatory role of FBXO6 in the control of ovarian cancer cells proliferation. We further test the migration and invasion ability of A2780 cells with or without FBXO6 expression. We found that in the absence of FBXO6, the migration and invasion abilities of A2780 cells were impaired (Fig. 4E–G). Furthermore, a nude mice model was used to investigate whether FBXO6 affects the oncogenic potential of ovarian cancer in vivo. To this end, BALB/c nude mice were subcutaneously injected with 1 × 10^7^ A2780 FBXO6^+/+^ or FBXO6^−/−^ cells, respectively. We found that KO of FBXO6 significantly decreased the tumor volume and tumor weight (Fig. 4H–J). Thus, these results indicate that FBXO6 plays a critical role in ovarian cancer cell proliferation, migration, and invasion.

### FBXO6 is overexpressed in ovarian cancer and negatively correlated with RNASET2

**Fig. 5:**
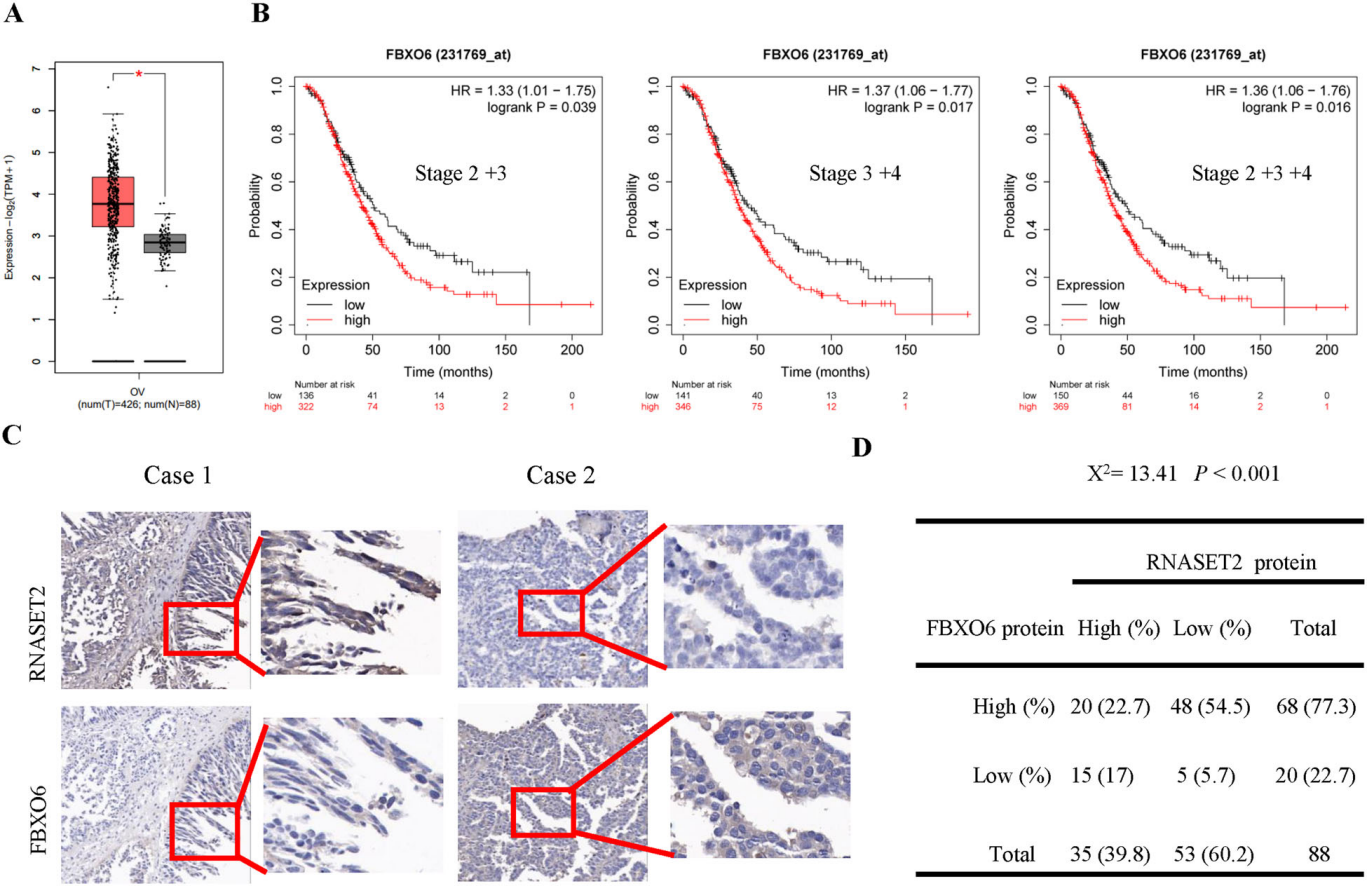
FBXO6 is overexpressed in ovarian cancer and negatively correlated with RNASET2. **A** The mRNA expression of FBXO6 between 426 ovarian cancer tissues and 88 non‐tumor tissues in TCGA database. **B** The overall survival (OS) curve of FBXO6 gene expression in ovarian cancer patients with advanced stages (http://kmplot.com/analysis/). **C** Immunohistochemical analyses of 88 specimens from ovarian cancer patients using anti-FBXO6 and anti-RNASET2 antibodies were performed. Representative images of IHC staining of tumors from two human ovarian cancer patients are presented. **D** The correlation study of FBXO6 and RNASET2 protein expression in 88 ovarian cancer samples is shown.

To understand the clinic relevance of FBXO6, we investigated its expression in ovarian cancer tissues. Analysis of The Cancer Genome Atlas dataset revealed that FBXO6 was significantly upregulated in ovarian cancer tissues (88 normal vs. 426 ovarian cancer, *p* < 0.05) (Fig. 5A). Next, we evaluated the prognosis value of FBXO6 in ovarian cancer patients using the online kmplotter database (http://kmplot.com/analysis/). Considering that the survival time of ovarian cancer patients at stage 1 is relatively longer than that of patients at advanced stages, the stage 1 patients were excluded from the analysis. A survival analysis of the clinical ovarian cancer database indicated a correlation between higher FBXO6 expression and the overall survival in ovarian cancer patients at advanced stages (stage 2 + 3, stage 3 + 4, or stage 2 + 3 + 4) were observed (Fig. 5B). Thus, these data suggested that aberrant expression of FBXO6 in advanced ovarian cancer patients is associated with poor prognosis. We next performed IHC analysis to evaluate the potential association between FBXO6 and RNASET2 in 88 human ovarian cancer specimens (Fig. 5C). The overexpression of FBXO6 in ovarian cancer samples (68/88, 77.3%) was confirmed by the IHC analysis (Fig. 5D). On the contrary, RNASET2 was downregulated in those ovarian cancer samples (53/88, 60.2%) (Fig. 5D). Moreover, a negative correlation between FBXO6 and RNASET2 proteins was observed in those ovarian cancer samples (*χ*^2^ = 13.41, *P* < 0.001) (Fig. 5D). Together, these results suggest that the downregulation of RNASET2 protein in ovarian cancer might be caused by FBXO6 overexpression.

### FBXO6 controls the progression of ovarian cancer at least in part by degrading RNASET2

**Fig. 6:**
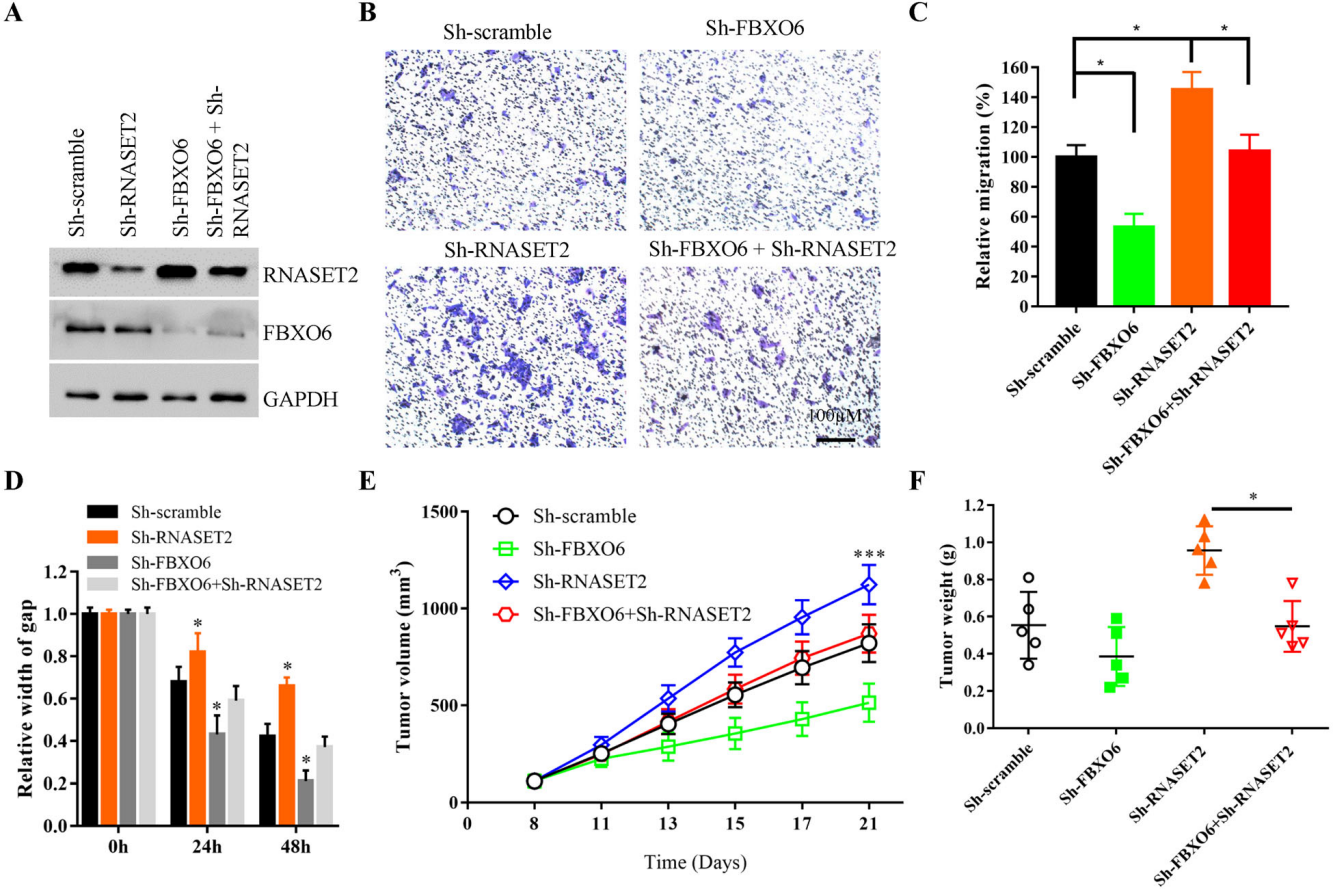
FBXO6 controls the progression of ovarian cancer at least in part by degrading RNASET2. **A** Western blot analysis of the WCLs derived from OVCAR-3 cells infected with the indicated lentiviral shRNA constructs. **B** Representative images of migrated OVCAR-3 cells infected with the indicated lentiviral shRNA constructs in a transwell assay with Matrigel. **C** Quantification of migrated cells in **B**. Data were shown as mean ± SD of three independent experiments. *p < 0.05. **D** The migration of OVCAR-3 cells infected with the indicated lentiviral shRNA constructs in scratch experiments. Data were shown as mean ± SD of three independent experiments. **p < 0.01. **E** OVCAR-3 cells (1 × 10^7^) infected with the indicated lentiviral shRNA constructs were subcutaneously injected into each nude mouse for about 3 weeks. Tumor volumes were detected at the indicated dates. *n* = 5 for each group. ***p < 0.001. **F** Tumor weights were measured after mice were killed. *p < 0.05.

We next investigated whether FBXO6 regulates ovarian cancer progress through RNASET2 degradation. First, we stably knocked down both *FBXO6* and *RNASET2* genes in OVCAR-3 cells by shRNAs. We found that shRNAs targeting RNASET2 can achieve about 70% knockdown efficiency of endogenous RNASET2 protein (Fig. 6A). Interestingly, the RNASET2 protein level in FBXO6 and RNASET2 double-silencing cells was close to that of nonspecific knockdown cells (Fig. 6A). This is probably caused by the increased protein stability of the residual-expressed RNASET2 protein. Through transwell and scratch experiments, we further confirmed that FBXO6 silencing significantly inhibited the migration and invasion ability of ovarian cancer cells. However, the impaired migration and invasion abilities of FBXO6-depleted ovarian cancer cells were largely restored when RNASET2 was simultaneously silenced (Fig. 6B–D), suggesting a functional interplay between FBXO6 and RNASET2 proteins in ovarian cancer cells. To further test this in vivo, we established xenografts using those cell lines in nude mice. Our results revealed that silencing the expression of RNASET2 in FBXO6-depleted ovarian cancer cells promoted tumor growth in vivo (Fig. 6E, F). Therefore, these results indicate that the oncogenic effect of FBXO6 in ovarian cancer is partly dependent on the degradation of RNASET2.

## Discussion

Previously, we have reported that FBXO6 could inhibit the endoplasmic reticulum stress-induced apoptosis by targeting glycosylated Ero1L for destruction^18^. Furthermore, we also showed that FBXO6 could inactivate the spindle checkpoint to generate multinucleated cells and accelerate cell cycle progress^16^. Therefore, these results indicate that FBXO6 might have certain characteristics of oncogenes. In the present study, we further revealed an oncogenic role of FBXO6 in ovarian cancer by targeting tumor suppressor RNASET2 for destruction. Our major discoveries include the following: (1) discovered previously unappreciated oncogenic roles of FBXO6, thus revealing a new role of FBXO6 in the development of ovarian cancer; (2) FBXO6 directly interacted with RNASET2 to target it for ubiquitin-dependent degradation; (3) FBXO6 deficiency stabilized RNASET2 protein to prevent ovarian cancer proliferation, migration, and invasion; (4) FBXO6 was overexpressed in clinical ovarian cancer tissues and high expression of FBXO6 was associated with poor survival of ovarian cancer patients at advanced stages.

The *RNASET2* gene is the only human member of the extracellular ribonuclease gene family. Unlike T1 ribonucleases, RNASET2 is widely expressed in most organisms and is related to stress response and host defense^6^. The human *RNASET2* gene deficiency is associated with infant cystic leukoencephalopathy, which causes psychomotor impairment, spasticity, and epilepsy^24^. The polymorphisms of the human *RNASET2* gene have been identified as a risk factor for several autoimmune diseases by a number of GWAS (Genome-wide association study) assays^25,26^. Loss of RNASET2 function in zebrafish caused neurodegeneration due to the accumulation of non-degraded RNA in the lysosomes^27^. Depletion of RNASET2 in rats caused altered lysosomal function and potential defects in autophagy due to decreased levels of lysosome-associated membrane protein 2 and elevated acid phosphatase and β-*N*-acetylglucosaminidase activities^28^. RNASET2 KO rats exhibit hippocampal neuropathology and deficits in memory, and patients with RNASET2 deficiency also exhibited a severe neurodegeneration phenotype^28^. Importantly, decreased expression of RNASET2 is beneficial for ovarian cancer development and, independent of its enzymatic activity, is most likely related to activation of the innate immune response and modulation of extracellular matrix protein components. Despite these advances, little is known about how to regulate RNASET2 protein.

Our results not only reveal a regulatory mechanism of FBXO6 on RNASET2 protein but also uncover that the oncogenic effect of FBXO6 in ovarian cancer partly depends on the degradation of RNASET2. Given the critical roles of RNASET2 in inhibiting the development of ovarian cancer, specific RNASET2 agonists might produce some positive clinical effects. However, either RNASET2-specific agonists or FBXO6 inhibitors are still not available yet. We further found that there was a negative correlation between RNASET2 and FBXO6 protein in clinical ovarian cancer samples, suggesting that blocking the interaction between FBXO6 and RNASET2 might be a novel anti-ovarian cancer strategy.

FBXO6 is widely expressed in various human tissues and the regulation of RNASET2 by FBXO6 indicates that FBXO6 could also be involved in the biological processes regulated by RNASET2 protein. Interestingly, FBXO6 has been reported to play a role in antiviral immunity^29^, indicating FBOX6 might have broad biological functions. Thus, it is important to determine whether FBXO6 is also involved in regulating autoimmune diseases or neurodegenerative diseases. It is worth noting that FBXO2, another member of the FBA family, is associated with a number of neurodegenerative diseases by targeting various substrates for destruction^30,31^. Together, these reports indicate that FBXO6 might also be involved in the development of neurodegenerative diseases at least by regulating the stability of RNASET2 protein. Therefore, our results reveal a previously unknown FBXO6-RNASET2 axis, which may contribute to the development of ovarian cancer. Thus, based on these clues, we believe that inhibition of FBXO6 may represent an effective strategy for the treatment of ovarian cancer.
